# The Effect of Intravenous Anesthetics on Ischemia-Reperfusion Injury

**DOI:** 10.1155/2014/821513

**Published:** 2014-01-16

**Authors:** Ahmet Eroglu

**Affiliations:** Karadeniz Technical University, Anesthesiology and Intensive Care Medicine, 61000 Trabzon, Turkey

## Abstract

The effects of intravenous anesthetics on ischemia-reperfusion injury (IRI) have been investigated in both animals and clinical studies. The protective effects and the dosages of the intravenous anesthetics on IRI were discussed in this paper. The prevention of the tissue injury after the IRI was demonstrated with intravenous anesthetics in some studies. In the future, the studies should be focused on the dosage of the anesthetics related to diminishing the tissue injuries. Further studies might be required in order to investigate the effects of the anesthetics on molecular levels.

## 1. Introduction

Ischemia-reperfusion injury (IRI) can be resulted from many factors such as the release of free oxygen radicals and consecutive lipid peroxidation, cell death by apoptosis or necrosis, inflammatory cytokines, and damage to the microvasculature [1, 2]. Reactive oxygen species (ROS) that appear with reperfusion injury damage cellular structures through the process of the lipid peroxidation of cellular membranes and yield toxic metabolites such as malondialdehyde (MDA), that is, used as a sensitive marker of ischemia-reperfusion injury. There is a balance between ROS and the scavenging capacity of antioxidant enzymes. Therefore, the total antioxidant capacity (TAC) is a functional outcome of both the oxidation capacity and the consumption rate of antioxidants during oxidative stress [1, 2]. There are three time frames in which protection against ischemia-reperfusion injury can be induced: before ischemia occurs, during ischemia, and after the ischemia at the onset of reperfusion.

Klune and Tsung [3] reported in a review article that the mechanism of organ damage after IRI has been studied extensively and consists of complex interactions of multiple inflammatory pathways. The major contributors to IRI include production of reactive oxygen species, release of proinflammatory cytokines and chemokines, and activation of immune cells to promote inflammation and tissue damage. Recent research has focused on the mechanisms by which these immune responses are initially activated through signaling molecules and their cellular receptors. Thorough understanding of the pathophysiology of liver IRI may yield novel therapeutic strategies to reduce IRI and lead to improved clinical outcomes [3]. Both experimental and clinical studies focusing on the reduction of IRI report that tissue injury may be prevented through the use of anesthetic agents or anesthesia methods. A variety of investigations using experimental animals have shown that intravenous anesthetic agents have a protective effect against ischemia and reperfusion injuries [4].

## 2. Opioids (Morphine, Remifentanil, and Fentanyl)

Evidence has now accumulated that intravenous anesthetics and some narcotics may be cardioprotective [4]. The cardioprotective effects of opioid receptor agonists have been consistently demonstrated in different models of ischemia-reperfusion injury in vivo as well as in vitro [5].

Morphine, remifentanil, and fentanyl as opioids have been effectively used for anesthesia/analgesia regimens in some surgical procedures [6–8]. Neuraxial morphine given during a postischemic period had been reported to have the potential to exacerbate ischemic spinal cord injury [9]. However, it remains unknown whether synthetic opioids administered systemically exacerbate ischemic injury. Therefore, Shirasawa and colleagues sought to compare the damage of the spinal cord after transient spinal cord ischemia in rabbits anesthetized with three different regimens: isoflurane, fentanyl with isoflurane, and remifentanil with isoflurane. Their results suggested that neither i.v. fentanyl nor i.v. remifentanil added to 0.5 MAC isoflurane exacerbated ischemic spinal cord injury in rabbits when compared to 1 MAC isoflurane [10]. In an experimental study, the authors determined whether morphine, administered via either the i.v. or intrathecal routes, can ameliorate hepatic IRI in rats with either normal or cirrhotic livers. They also tested whether morphine-mediated hepatoprotection involves *μ*-opioid receptor activation and the PI3K/Akt and Jak2/STAT3 pathways as these pathways have previously been shown to be involved in morphine-mediated cardioprotection. In the study morphine as an opioid was administered either i.v. or intrathecally 10 min before initiating 1 h of ischemia followed by 6 h of reperfusion in normal rat liver. The authors reported that morphine preconditioning protects against IRI in both normal and cirrhotic rat livers. The mechanisms of morphine-induced hepato-protection are most likely multifactorial. These multifactorials involve opioid receptors, phosphatidylinositol-3-kinase, and Akt [11].

Remifentanil is a new, potent, ultra-short-acting selective opioid receptor agonist in the clinical use of anesthesia and analgesia [7, 12]. In an animal study, it was evaluated whether remifentanil was cardioprotective when administered in postconditioning fashion and compared its effect with that of ischemic postconditioning. The relative role of opioid receptor subtypes in both regimes was also investigated by the use of subtype-specific opioid receptor antagonists. Remifentanil postconditioning was evaluated using a 5 min infusion of the drug at 1, 5, 10, or 20 *μ*g/kg/min of body weight. The results of the study indicated that remifentanil postconditioning protected the heart from ischemia-reperfusion injury to a similar extent as of ischemic postconditioning. This protection involves *κ* and *δ* but not *μ* opioid receptor activation [13].

An experimental mice study had demonstrated that a single bolus of 1 *μ*g/kg of remifentanil given before tissue ischemia was protected against IRI in the small intestine, allowing us to bypass the inherent adverse effects of conventional *μ*-opiods such as persistent inhibition of gastrointestinal motility and respiratory drive. A marked amelioration of mucosal injury in remifentanil-treated mice was accompanied by a reduction of oxidative stress locally and inflammation systemically, as evidenced by decreased concentrations of gut tissue MDA and plasma IL-6 [14].

Another study determined the effects of remifentanil in focal brain ischemia and ischemia-reperfusion injury. Mechanisms linked to mitogen-activated protein kinases, including extracellular signaling-regulated kinase (ERK) 1/2, p38 kinases, and c-Jun N-terminal kinase (JNK), and various cytokines were also examined. Remifentanil (5 *μ*g/kg/min) was given alone or combined with naltrindole (*δ*-opioid receptor antagonist; 1 mg/kg). Remifentanil infusion was started 10 minutes before middle cerebral artery occlusion (MCAO) and continued throughout. It was concluded from the study that remifentanil may be neuroprotective against focal ischemia-reperfusion injury, possibly through the activation of *δ*-opioid receptors and attenuation of ERK 1/2 activity and TNF-*α* production, in the rat brain [15].

Fentanyl, a synthetic derivative of morphine, is widely used for patients undergoing cardiovascular surgeries. Clinical and experimental evidences suggest that most of the cardiovascular effects of fentanyl are mediated by opioid receptors (ORs) acting. In an experimental study, the cardioprotective effects of IV-administered fentanyl using a model of myocardial ischemia-reperfusion injury associated with pharmacologically induced central sympathetic over activity were investigated. The results of the study concluded that fentanyl's effects for limiting myocardial ischemic injury are mediated via peripheral ORs, while opioid's antiarrhythmic actions are mediated via central OR agonism [16].

## 3. *α*-2*a* Adrenergic Agonists (Dexmedetomidine)

Dexmedetomidine, a potent and highly selective *α*-2 adrenoreceptor agonist, is widely used for sedation in intensive care units (ICU). Dexmedetomidine also offers good perioperative hemodynamic stability and an intraoperative anesthetic sparing effect [17]. The results of an experimental study clearly demonstrated that oxidative stress parameters were significantly altered in experimental hepatic IR injury in the rats. Dexmedetomidine was found to be a protective agent against the oxidative alterations in hepatic IR injury on the liver and remote organs, when given before induction of ischemia. Moreover, dexmedetomidine protected against the harmful effects of IR in terms of the histopathological changes in the liver. Therefore, dexmedetomidine may be used as an adjuvant anesthetic agent before surgery for patients with potential hepatic IR injury [18].

Yagmurdur and colleagues examined the effect of dexmedetomidine on ischemia-reperfusion injury due to tourniquet application during upper-extremity surgery by determining blood malondialdehyde and hypoxanthine levels. Alterations in aspartate aminotransferase, alanine aminotransferase, creatine phosphokinase, lactate dehydrogenase, uric acid, and creatinine levels were also assessed. In the dexmedetomidine group, a continuous infusion of dexmedetomidine (1 *μ*g/kg for 10 minutes, followed by 0.5 *μ*g/kg h^−1^) was used until the end of surgery, whereas the control group received an equivalent volume of saline. Their results suggest that dexmedetomidine may offer advantages by inhibiting lipid peroxidation in the case of anticipated ischemia-reperfusion injury, which would occur in upper-extremity surgery requiring tourniquet application [19].

A clinical study evaluated the effects of dexmedetomidine on tourniquet-induced ischemia-reperfusion injury during general anesthesia by measuring MDA and TAC levels when dexmedetomidine was added to the general anesthesia. The main findings of the study demonstrated that serum MDA levels were decreased when compared to basal values at 5 and 20 minutes ATR and that TAC was lower than basal values at 1 minute before and at 5 minutes ATR and reached the basal level at 20 minutes ATR. However, these findings were similar to the results obtained from the group that was not given dexmedetomidine [20].

Dexmedetomidine has been used for purposes of anesthesia and sedation, and experimental studies have demonstrated its neuroprotective effects. However, it has also been shown that the constriction of cerebral vessels in response to high doses of dexmedetomidine decreases cerebral blood flow. A study tested the hypothesis that dexmedetomidine-induced cerebral hypoperfusion exacerbates ischemic cerebral injury. The effects of dexmedetomidine on cerebral blood flow and mean arterial blood pressure were studied first in this study. Six rats received intravenous infusions of dexmedetomidine in doses ranging from 0.01 to 10 *μ*g/kg min^−1^. Hypertension following the administration of high-dose dexmedetomidine is associated with cerebral hypoperfusion and the exacerbation of ischemic brain injury, possibly through alpha-2-induced cerebral vasoconstriction [21].

## 4. Propofol

Propofol is a rapidly acting intravenous hypnotic agent, that is, frequently used in clinical anesthesia administrations [7, 22, 23]. Propofol is an intravenous anesthetic with neuroprotective effects against cerebral ischemia-reperfusion injury [24]. Few studies regarding the neuroprotective and neurobehavioral effects of propofol have been conducted, and the underlying mechanisms are still unclear. Because IRI may result in neuronal apoptosis, the apoptosis regulatory genes B-cell leukemia-2 (Bcl-2) and Bcl-2-associated X protein (Bax) may be involved in the neuroprotective process. In a study, cerebral ischemia was induced by clamping the bilateral common carotid arteries for 10 min. Propofol (1.0 mg/kg/min) was administered intravenously for 1 h before the induction of ischemia. The results of this study showed that neurobehavioral scores were higher in propofol-treated rats compared to ischemia-reperfusion injury-induced rats with no propofol treatment. Moreover, the hippocampal expression of Bcl-2 was significantly higher, while the expression of Bax was significantly lower in propofol-treated rats compared to IRI-induced rats at 24 h after ischemia. Hence, this study suggests that the neuroprotective effects of propofol against neuronal apoptosis may be a consequence of the regulation of Bcl-2 and Bax [25].

Remote pulmonary injuries after hepatic reperfusion are frequently caused by reactive oxygen species (ROS) induced damage. The choice of anesthetics may affect the balance between oxidants and antioxidants, and propofol, a commonly used anesthetic, has an antioxidant effect. In a study a model was developed to study the effect of propofol on pulmonary function with hepatic ischemia-reperfusion injury manipulation. The aim of the study was to determine remote pulmonary dysfunction after hepatic reperfusion and determine if propofol affects this dysfunction by altering ROS production from the liver or lungs. Remote pulmonary dysfunction and reperfusion injury in the liver were demonstrated in this rat model, as well as massive ROS production and lipid peroxidation. As a conclusion of this study, propofol infusion attenuated remote pulmonary injury by lessening oxidative injury from the reperfused liver [26].

In an another clinical study, it was demonstrated that total intravenous anesthesia with propofol and regional anesthesia techniques provided better antioxidant defense and reduced endothelial dysfunction than general inhalational anesthesia with sevoflurane during tourniquet application in pediatric extremity surgery [27].

The antioxidant properties of propofol have been shown to improve ischemia-reperfusion injury. In an animal study, the authors investigated whether anesthesia with propofol can ameliorate remote lung injury induced by intestinal ischemia-reperfusion (IIR) in rats. Using propofol to induce and maintain anesthesia efficiently prevented IIR-induced lung injury. Systemic antioxidant protection, improvement of intestinal injury, inhibition of the inflammatory response, and preservation of the alveolar-capillary permeability seem to be crucial, mediating mechanisms for this simple and clinically relevant intervention [28].

## 5. Ketamine

Ketamine is an anesthetic with anti-inflammatory properties, which has shown protective effects on IRI in various organs [29, 30]. An experimental study investigated the effects of ketamine on intestinal IRI. Male Wistar rats underwent either sham surgery or 30 min of intestinal ischemia followed by 60 min of reperfusion. Ketamine pretreatment was administered by intraperitoneal injections at doses of 100, 50, 12.5, or 6.25 mg/kg. The intestinal morphology, mucosal damage, leukocyte infiltration, serum P-selectin, serum intracellular adhesion molecule-1 (ICAM-1), serum antithrombin-III (ATIII), and myenteric ganglion cell structure were evaluated. Intestinal IRI led to severe mucosal damage, leukocyte (especially neutrophil) infiltration, P-selectin and ICAM-1 elevations, ATIII depletion, and myenteric ganglion cell morphological alterations. According to the results of this study, the ketamine dose dependently diminished these alterations (except for ICAM-1 serum levels), reaching statistical significance at 100, 50, and 12.5 mg/kg. Ketamine protects the intestine against ischemia-reperfusion injury [29].

In an another experimental study, the authors sought to determine whether a higher dose of ketamine-xylazine (KX) protected isolated guinea pig hearts against myocardial ischemia-reperfusion injury. Male Hartley guinea pigs (Crl : HA; 275 to 300 g; *n* = 14) were anesthetized with either of the 2 doses of KX (K: 85 mg/kg, X: 15 mg/kg; or K: 200 mg/kg, X: 60 mg/kg). They found that higher doses of KX used to anesthetize guinea pigs led to reduction in myocardial infarct size and improved hemodynamic function after experimental ischemia-reperfusion. These results suggest that supplementation of KX to ensure an adequate anesthetic plane may introduce unwanted variability in ischemia-reperfusion studies [30].

The effect of ketamine on IRI was compared to other intravenous anesthetics. The aim of the study was to investigate the effects of ketamine and pentobarbital anesthesia on the motility alterations and tissue injury caused by ischemia-reperfusion injury in rats. In the study rats were anesthetized with either pentobarbital sodium (50 mg/kg) or ketamine (100 mg/kg). The results of the study showed that ketamine anesthesia is associated with diminished intestinal injury and abolishes the intestinal transit delay induced by ischemia-reperfusion injury [31].

Propofol and ketamine were compared in an animal study. This study examined the cardioprotective effects of propofol and ketamine with and without N-acetylcysteine (NAC). Creatine kinase (CK), myocardial band of creatine kinase (CK-MB), and troponin-I (Tn-I) levels CK, CK-MB, and Tn-I levels did not differ significantly between the ketamine groups (1–3) and the propofol groups (4–6) *P* > .05). Malondialdehyde levels in the propofol groups (4–6) were significantly lower than in the ketamine groups (1–3; *P* < .05). In this rat model of global cardiac ischemia, propofol with NAC attenuates myocardial injury more than ketamine (with or without NAC) [32].

## 6. Barbiturates (Thiopental and Pentobarbital), Etomidate, and Midazolam

Pentobarbital and thiopental are barbituric acid derived, sedative acting anesthetic agents. Dogan and colleagues reported that thiopental and propofol, especially thiopental, are more effective to protect renal ischemia-reperfusion injury in an experimental study [33]. Another study was demonstrated to compare the influence of three anesthetics (60 mg/kg thiopental, 1200 mg/kg urethane, and 60 mg/kg pentobarbital intraperitoneally) on ventricular arrhythmias and to combine it with measured hemodynamic parameters to find the most suitable agent for such experiments. In the model of ischemia- and reperfusion-induced arrhythmias in Sprague-Dawley rats, after left anterior descending coronary artery occlusion (7 minutes) and reperfusion (15 minutes), the following parameters had been measured or calculated: mortality index; ventricular fibrillation and tachycardia incidence and duration; systolic, diastolic, and mean arterial blood pressure; heart rate; myocardial index of oxygen consumption; and plasma creatine kinase concentration. According the results of this study, pentobarbital is the most suitable anesthetic offering stable hemodynamic values during arrhythmia studies. These hemodynamic values were similar to physiological values in awake rats; the long arrhythmia duration during reperfusion and approximately 50% mortality index are crucial parameters for evaluating antiarrhythmic drugs [34].

Etomidate, commonly used for cardiac patients, is an intravenous anesthetic agent. Etomidate, thiopental, propofol, and ketamine were compared in a study. The aim of the study was to compare the use of different anesthetic drugs in a skeletal IRI model. Rats in each group were anesthetized either with thiopental, ketamine, propofol, or etomidate. Malondialdehyde, superoxide dismutase, catalase, and glutathione peroxidase were measured in skeletal muscle via a spectrophotometer. In this study rats anesthetized with ketamine (60 mg/kg), propofol (100 mg/kg), or etomidate (20 mg/kg) did not show increased malondialdehyde levels in comparison to control levels. While the drugs did not cause a distinction in the levels of superoxide dismutase, catalase, glutathion peroxidase, iron, and copper, zinc was in a lower level in IRI group compared to sham control. It was concluded that ketamine, propofol, and etomidate, with anesthetic doses, denoted efficacious effects on IRI; hence the drugs might be preferred in certain operations with the risk of IRI [35].

Interest in the doses of multiple anesthetics in ischemia-reperfusion injury has studied. Etomidate, thiopental, propofol, and intralipid were compared in a study. The purpose of this study was to investigate and compare the efficiency of ketamine, thiopental, propofol, etomidate, and intralipid in reducing the injury induced by free radicals in a rat model of renal IRI. Rats in the IRI groups were given ketamine (20 mg/kg), thiopental (20 mg/kg) propofol (25 mg/kg), etomidate (10 mg/kg), and 10% of intralipid (250 mg/kg) intraperitoneally 15 min prior to the ischemia for 60 min, followed by reperfusion for 60 min. Biochemical malondialdehyde (MDA), superoxide dismutase (SOD), catalase (CAT), blood urea nitrogen (BUN), creatine (Cr), and aspartate aminotransferase (AST) were determined, and histopathological analysis was performed with these samples. MDA levels were lower in the ketamine, thiopental, and propofol groups compared to the control group (*P* < 0.05). In the thiopental and propofol groups, the levels of histopathological scores were significantly lower than control and etomidate groups in ischemia-reperfusion injury. These results demonstrated that IRI was significantly reduced in the presence of propofol and thiopental. The protective effects of these drugs may belong to their antioxidant properties. These results may indicate that propofol and thiopental anesthesia protects against functional, biochemical, and morphological damage better than control in renal ischemia-reperfusion injury [36].

Erturk and colleagues compared the effects of propofol and N-acetyl cysteine (NAC) on tourniquet-induced ischemia-reperfusion injury by determining malonyldialdehyde, ischemia-modified albumin, lactate, blood gas, and hemodynamic levels in arthroscopic knee surgery. Sixty ASA I or II patients were randomized into three groups. Intrathecal anesthesia was administered using 0.5% heavy bupivacaine in all patients. In group P, propofol was administered in a 0.2 mg kg^−1^ bolus, followed by infusion at a rate of 2 mg kg^−1^ h^−1^; in group NAC, NAC was administered as an infusion at a rate of 5 mg kg^−1^ h^−1^, and, in group C (the control group), an equal volume of isotonic saline was administered in patients until 30 min after reperfusion. Plasma concentrations of malonyldialdehyde, ischemia-modified albumin, and lactate in groups P and NAC were significantly lower than those in group C. The author reported that comparisons of groups P and NAC revealed no significant differences. Small-dose infusions of both propofol and NAC appear to provide similar protection against ischemia-reperfusion injury in arthroscopic knee surgery [37].

Midazolam, a benzodiazepine, is used for sedation anesthesia in some surgical procedures [22, 23]. An animal study compared the neuroprotective effects of propofol, thiopental, etomidate, and midazolam as anesthetic drugs in fetal rat brain in the ischemia-reperfusion (IR) model. In the study pregnant rats of day 19 were randomly allocated into eight groups. Fetal brain ischemia was induced by clamping the utero-ovarian artery bilaterally for 30 min and reperfusion was achieved by removing the clamps for 60 min. One milliliter intralipid solution, 40 mg/kg propofol, 3 mg/kg thiopental, 0.1 mg/kg etomidate, and 3 mg/kg midazolam were administered intraperitoneally in the vehicle group, propofol group, thiopental group, etomidate group, and midazolam group, respectively, 20 min before IR procedure. The results of the study reported that the anesthetic drugs including propofol, thiopental, etomidate, and midazolam decrease the lipid peroxidation back to control values, while only propofol and midazolam have a protective effect on mitochondrial damage [38].

## 7. Local Anesthetics (Lidocaine)

Lidocaine is a widely used local anesthetic and antiarrhythmic agent that has been shown to have cardioprotective effects against myocardial ischemia and reperfusion injury by blocking cardiac sodium channels, reducing intracellular calcium loading, reducing ROS production, and modulating mitochondrial bioenergetics [39]. In a randomized, double-blinded trial, 99 patients received i.v. lidocaine 2% (i.e., a 1.5 mg kg^−1^ bolus at induction of anesthesia followed by a 2.0 mg kg^−1 ^h^−1^ infusion intraoperatively) or an equal volume of saline. Serum creatine kinase-myocardial band (CK-MB) and troponin I (TnI) concentrations were measured before surgery. The result of this study found that the continuous i.v. infusion of lidocaine during surgery reduced myocardial injury in patients undergoing off-pump coronary artery bypass graft surgery [40].

Interest in the dosage of anesthetics in skeletal ischemia-reperfusion injury has recently increased. Thus, the aim of the study was to compare the effects of subanesthetic doses of ketamine, propofol, and etomidate on a skeletal IRI model. The results of the study showed that subanesthetic doses of ketamine, propofol, and etomidate displayed beneficial effects in IRI [41].The dosage studies of the intravenous anesthetics on IRI were shown in Table 1.

In a conclusion, the prevention of the tissue injury after the IRI was demonstrated with most of the intravenous anesthetics (e.g., propofol, midazolam, and thiopental) in both animal and clinical studies. In the future, the studies should be focused on the dosage of the anesthetics related to diminishing the tissue injuries. Further studies might be required in order to investigate the effects of the anesthetics on molecular levels.

## Figures and Tables

**Table 1:** The dosage studies of the intravenous anesthetics on IRI.

Anesthetic	Dosage	Clinical/animal	Organ/tissue	Marker	IRI
Morphine	(i) 1–100 *µ*g/kg (ii) 30 *µ*g	(i) Animal (11) (ii) Animal (9)	(i) Liver (ii) Spinal cord	(i) Opioid receptors (ii) Opioid receptors	(i) Protect (ii) Exacerbate

Fentanyl	5 or 50 *µ*g/kg,	Animal (16)	Myocard	Opioid receptors	Protect

Remifentanil	(i) 1 *μ*g/kg (ii) 5 *μ*g/kg/min (iii) 1, 5, 10 or 20 *µ*g/kg/min	(i) Animal (14) (ii) Animal (15) (iii) Animal (13)	(i) Small intestine (ii) Brain (iii) Heart	(i) MDA, IL-6 (ii) ERK, TNF-*α* (iii) Opioid receptors	(i) Protect (ii) Protect (iii) Protect

Dexmedetomidine	(i) 1 microg/kg for 10 minutes, followed by 0.5 microg kg^−1^ h^−1^ (iii) 100 *μ*g/kg	(i) Clinical (19) (ii) Animal (18)	(i) Upperextremity (ii) Liver	(i) MDA (ii) No	(i) Protect (ii) Protect

Propofol	(i) 25 mg/kg/h (ii) 25 mg/kg (iii) 0.2 mg kg^−1^ bolus, followed by infusion at a rate of 2 mg kg^−1^ h^−1^	(i) Animal (26) (ii) Animal (36) (iii) Clinical (37)	(i) Pulmonary (ii) Renal (iii) Knee surgery	(i) No (ii) MDA (iii) MDA, IMA	(i) Protect (ii) Protect (iii) Protect

Ketamine	(i) 85–200 mg/kg (ii) 100 mg/kg	(i) Animal (30) (ii) Animal (29)	(i) Heart (ii) Intestinal	(i) No (ii) No	(i) Protect (ii) Protect

Thiopental	60 mg/kg	Animal (34)	Coronary	No	Protect

Pentobarbital	60 mg/kg	Animal (34)	Coronary	No	Protect

Etomidate	(i) 20 mg/kg (ii) 10 mg/kg	(i) Animal (35) (ii) Animal (36)	(i) Skeletal muscle (ii) Renal	(i) MDA (ii) MDA	(i) Protect (ii) Protect

Midazolam	3 mg/kg	Animal (38)	Mitochondrial	No	Protect

Lidocaine	1.5 mg kg^−1^ bolus and 2.0 mg kg^−1^ h^−1^ infusion	Clinical (40)	Myocardial	CK-MB, TnI	Protect
